# Effects of olanexidine gluconate on preoperative skin preparation: an experimental study in cynomolgus monkeys

**DOI:** 10.1099/jmm.0.000462

**Published:** 2017-05-18

**Authors:** Hikaru Nakata, Yoshie Tsubotani, Takuya Nii, Akifumi Hagi, Yasuhide Inoue, Tadashi Imamura

**Affiliations:** ^1^​ Naruto Research Institute, Research and Development Center, Otsuka Pharmaceutical Factory, Inc., Tokushima, Japan; ^2^​ Ina Research Inc., Nagano, Japan

**Keywords:** olanexidine, antiseptic, bactericidal effect, *in vivo* model, cynomolgus monkey

## Abstract

**Purpose:**

To determine the bactericidal efficacy of a new topical antiseptic for preoperative skin preparation, olanexidine gluconate (development code: OPB-2045G), against transient or resident bacterial flora on the skin of cynomolgus monkeys.

**Methodology:**

After measuring baseline bacterial counts on test sites marked on the abdomens, we applied olanexidine, chlorhexidine or povidone–iodine. After 10 min (fast-acting effect) and 6 h (long-lasting effect), bacterial counts were measured again and log_10_ reductions were calculated. In addition, we determined the bactericidal effects on the skin contaminated with blood before or after applying the antiseptics.

**Results:**

In the non-blood-contaminated condition, the mean log_10_ reductions of olanexidine at doses of 1–2 % were significantly higher than those of saline (negative control), but did not significantly differ from those of 0.5 % chlorhexidine and 10 % povidone–iodine at either time point. But olanexidine was significantly more effective at both time points than chlorhexidine and povidone–iodine when applied after the site was contaminated with blood. Olanexidine was also significantly more effective than chlorhexidine and as effective as or more effective than povidone-iodine at both time points when skin was contaminated with blood after the antiseptics were applied.

**Conclusion:**

The bactericidal effects of olanexidine were comparable to those of commercial antiseptics such as chlorhexidine and povidone–iodine in non-blood-contaminated conditions. More importantly, the effect of olanexidine was hardly affected by blood unlike commercial antiseptics. Thus, it is considered that olanexidine has a favourable property for skin preparation in various types of surgical treatments.

## Introduction

Surgical site infections are a major problem in healthcare delivery. These infections can increase the length of hospital stays, medical costs and the risk of death. Topical antiseptics applied preoperatively can reduce the risks of such infections. However, povidone–iodine (PVP-I) and chlorhexidine gluconate (CHG), two of the most commonly used topical antiseptics, are not always effective. It is well known that organic substances (e.g. blood or sputum) significantly decrease the antimicrobial activity of PVP-I [1] and the efficacy of PVP-I on enterococci, including vancomycin-resistant enterococci (VRE), is reportedly low [2, 3]. Although CHG has a broad spectrum of microbicidal activity, it still may not be sufficient to eradicate methicillin-resistant *Staphylococcus aureus* (MRSA) and VRE [4, 5]. New antiseptics were expected to address these problems, but only a few new agents have been introduced in the past 50 years.

**Fig. 1.**
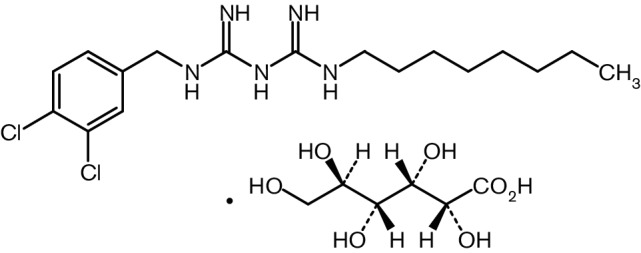
Chemical structure of olanexidine gluconate.

We developed a new bactericidal biguanide compound, olanexidine gluconate [1-(3,4-dichlorobenzyl)-5-octylbiguanide mono-d-gluconate (OLG); Fig. 1]. The efficacy of OLG against MRSA and VRE in both *in vitro* and *in vivo* animal models is higher than that of CHG and PVP-I [6], and OLG has a broad spectrum of antibacterial activity against bacterial strains, including clinical isolates [7]. The bactericidal mechanism of OLG differs from that of a similar biguanide compound, CHG [7]. Moreover, clinical trials of the topical formulation containing 1.5 % OLG were completed with favourable results, and it is now used clinically in Japan.

Generally, the efficacy of an antiseptic is evaluated in *in vitro* non-clinical studies, such as suspension tests and carrier tests. The effects of organic substances that can interfere with antimicrobial activity (e.g. blood) also have been tested *in vitro* [8, 9]. Some *in vitro* microbiological studies use animal models; for example, a pig skin model has been used to mimic human skin conditions [10, 11]. However, few *in vivo* studies using animal models have been reported [12]. Reliable animal models are required not only for correctly evaluating the efficacy of antiseptics in explanatory trials, but also for evaluating their clinical effectiveness in pragmatic trials. Although primate models for evaluating the efficacy of hand hygiene have been reported [13, 14], we found no *in vivo* studies that evaluated the effectiveness of a topical antiseptic in monkeys. We developed a primate model using cynomolgus monkeys (*Macaca fascicularis*), which is useful for evaluating developmental antiseptics that cannot yet be tested in humans and in non-clinical studies of clinical application methods and specific clinical conditions, such as skin contamination with blood or other fluids. Using this model, we assessed the bactericidal efficacy of olanexidine as a topical antiseptic.

## Methods

### Test antiseptics and materials

The Olanedine antiseptic solution (Otsuka Pharmaceutical Factory), StericlonW solution 0.5  (Kenei Pharmaceutical), and Isodine solution 10 % (Meiji Seika Pharma) were used as OLG, 0.5 % CHG and 10 % PVP-I, respectively.

The following bacterial counting reagents containing neutralizers were used: sampling solution [74 mmol l^−1^ phosphate buffer solution (pH 7.8) containing 0.1 % Triton X-100, 10 % polysorbate 80, 2 % lecithin (soybean), 5 % polyoxyethylene cetyl ether and 0.5 % sodium thiosulfate hydrate], diluting fluid [Butterfield’s phosphate buffer containing 10 % polysorbate 80, 2 % lecithin (soybean), 5 % polyoxyethylene cetyl ether and 0.5 % sodium thiosulfate hydrate] and tryptic soy agar (TSA) medium (Difco, Becton Dickinson; 40 g l^−1^) containing 1 % polysorbate 80, 0.12 % lecithin (soybean), 0.1 % Tamol NN8906 and 0.05 % sodium thiosulfate hydrate as neutralizers (TSA+).

A neutralization study was performed to ensure that the neutralizers used in the bacterial counting agents effectively quenched the antimicrobial activity of the test antiseptics and were not toxic to the test bacteria. Study procedures were based on ASTM E1054-08 (*Standard Test Methods for Evaluation of Inactivators of Antimicrobial Agents*) [15]. *Staphylococcus epidermidis* (ATCC 12228), *Staphylococcus aureus* (ATCC 6538) and *Acinetobacter baumannii* (ATCC BAA-747) were used as the test bacteria in the neutralization study. Bacterial strains were purchased from Microbiologics.

### Animals

Male cynomolgus monkeys imported from the Philippines, China or Vietnam were individually housed in stainless steel cages with high-pressure melamine-faced plate walls (48W×85D×80 cm H, with stainless steel play equipment for enrichment). The room was maintained at about 25 °C, between 40 to 80 % humidity and had a 12 h light/dark cycle (7 : 00 AM to 7 : 00 PM). Approximately 100 g of monkey chow (PS-A; Oriental Yeast) was given every day and drinking water was available *ad libitum* through an automatic water system in each cage.

Two test sites, right and left, were established on the abdomen of each monkey. Different antiseptics were tested at each site. To reduce the variation in baseline bacterial counts and to ensure the baseline bacterial counts were sufficient to evaluate the bactericidal effects of antiseptics, only animals having bacterial counts of 4.0 to 5.5 log_10_ (c.f.u. cm^−2^) in the groin were used. Animals were assigned to a test group so that no inter-group bias was present regarding the bacterial count in the groin.

### Bacteria sampling and counting

Bacteria samples were obtained from the skin of monkeys under anaesthesia using the cylinder sampling technique, as described previously [16]. Briefly, a sterile metal cylinder (2.1 cm, inner diameter) was pressed against the skin and 2.5 ml of sampling solution was poured into the cylinder. After massaging the surface of the skin inside the cylinder with a sterile rubber policeman for 1 min, the solution was collected in a pipette and the process was repeated with a fresh 2.5 ml charge of sampling solution. The two samples were combined and serially diluted with diluting fluid. Duplicate cultures were prepared from each dilution by the agar pour-plate culture method using the TSA+. Plates were incubated at 34.7 to 37.1 °C for 38 to 51 h and the number of c.f.u. was counted in each plate after incubation. When the mean of the count from the duplicate plates was one or less, the value was set to 1 c.f.u. cm^−2^ so that a zero value would result after log transformation.

### Experiment 1: the clean antisepsis condition

The purpose of experiment 1 was to determine the bactericidal effects of OLG applied to normal skin without any treatment to simulate a standard pre-surgical application (hereafter, this condition is refered to as the ‘clean condition’). The 24 animals were anaesthetized with an intramuscular ketamine/xylazine mixture and placed supine. The hair on the abdomen was removed using clippers and two test sites were established: one on each side of the abdomen. Three sampling areas (3.5×3.5 cm each) arranged vertically on each test site were marked using an indelible felt-tip pen. Each test site of each animal was allocated to one of the test antiseptics. Each sampling area at each test site was allocated to one of three sampling times: baseline, 10 min or 6 h after application of the test antiseptics [17].

Bacteria samples were collected from the baseline bacteria sampling area by the cylinder sampling technique described above. In this experiment, 1 % OLG, 1.5 % OLG, 2 % OLG, 0.5 % CHG, 10 % PVP-I and normal saline (as a negative control) were used as test antiseptics. Two sponges with handles (Livedo) were immersed in the antiseptic. The first sponge was pressed against the test site by hand. The sponge was moved in a direction from the animal’s head to foot and vice versa for 30 s to apply the antiseptic evenly over the test site. The sponge was turned over and the antiseptic was further applied for another 30 s in a similar manner. The second sponge was applied in the same way as the first, thus providing a total of 2 min of application, 1 min for each sponge.

Bacterial samples were collected 10 min after application. Then, the sampling areas were covered with sterile gauze and secured with tape (PetFlex, a flexible cohesive bandage; Andover Healthcare). In addition, the animals wore animal jackets to protect the test sites. The gauze was removed from the test site 6 h after application and bacteria samples were immediately collected as before.

To identify the bacteria, colonies were selected without known bias from the plates used to determine the baseline counts and newly subcultured on the TSA plates. The plates were incubated at 35 °C until colony formation. A single colony was Gram-stained, observed microscopically and subjected to the catalase reaction. From the Gram-reaction, morphology, and the presence or absence of catalase, the test bacteria were identified using the API kit for manual identification of micro-organisms (API Staph or API Coryne; Sysmex bioMérieux). The results of identification were judged using apiweb identification software (Sysmex bioMérieux).

### Experiment 2: the trauma antisepsis condition (dirty condition)

In this experiment, the bactericidal effects of the antiseptics were examined for skin contaminated with blood before application, to model a traumatic injury. Blood samples were obtained from 48 animals not involved in assessing bactericidal effects. Each animal was anaesthetized with the intramuscular ketamine/xylazine mixture. Blood (10 ml) was collected from the veins of the hind limbs using a sterile syringe and sterile needle and pooled.

The 12 animals used to evaluate bactericidal effects were anaesthetized, and test sites and bacterial sampling areas on abdomens were marked as in experiment 1. The pooled blood (20 ml per test site) was spread on the test sites using a sterile syringe and a sterile bacteria spreader. Bacteria samples were then collected from the baseline sampling areas by the cylinder sampling technique. In this experiment, 1.5 % OLG, 0.5 % CHG and 10 % PVP-I were applied as follows. The OLG was applied as in experiment 1, with sponges. The other antiseptics were applied with a sterile cotton ball immersed in each antiseptic with a sterile forceps held by hand. Three cotton balls containing the antiseptic were prepared for each test site. One at a time, each of the three cotton balls was pressed against the test site with appropriate pressure using a sterile forceps and applied in a series of separate, concentric circles, as opposed to an expanding spiral motion. Bacteria samples were collected from the sampling areas 10 min and 6 h after antiseptic application, as described.

### Experiment 3: the post-application bleeding condition (dirty condition)

In the third experiment, the bactericidal effects were examined for skin contaminated with blood after application of the antiseptic to model bleeding after skin preparation. The animals were prepared as in experiment 1. Bacteria sampling and blood and antiseptic application were carried out as in experiment 2.

Bacteria samples were collected from the baseline sampling area. After applying the test antiseptic as in experiment 2, blood was applied (20 ml per test site) on the test sites. The bacteria samples were collected from the specified sampling areas at 10 min and 6 h after application as described.

### Statistical methods

Data were analysed by the Tukey’s multiple comparisons test at the same time point. Alpha was set at 0.05. Data were analysed with the SAS 9.2 software program (SAS Institute Japan) and EXSUS 7.7 software (CAC Exicare Corporation).

### Ethical approval

All animal experiments were conducted at Ina Research Inc. (Nagano, Japan) in compliance with the Partial Amendments to the Law for the Humane Treatment and Management of Animals (Law No. 68, June 22, 2005, Japan), and the Guidance for Animal Care and Use of Ina Research Inc., and in accordance with protocols reviewed by the Institutional Animal Care and Use Committee of Ina Research Inc., which is fully accredited by AAALAC International (Accredited Unit no. 001107).

## Results

### Baseline measurements

In the preliminary experiment, we measured the viable bacterial count in the abdomen and groin of the cynomolgus monkey. In 45 animals, the bacterial counts on the abdomen and groin [log_10_ (c.f.u. cm^−2^); mean±sd] were 4.13±0.56 (range, 3.01 to 5.58) and 4.27±0.59 (range, 2.39 to 5.46), respectively. The correlation between the two bacterial counts (correlation constant, *r*) was 0.77, suggesting a good correlation. We also compared the viable bacterial counts between the left and right side of each abdomen. The 95 % confidence interval (CI) of the difference of viable bacterial counts [log_10_ (c.f.u. cm^−2^), 64 animals] between the left and right test sites was −0.012 to 0.169, which contained zero, indicating no statistically significant difference.

In these preliminary experiments, the inter-animal difference in bacterial counts was expected to be large, but the inter-sampling site differences (e.g. between abdomen and groin, or left and right at abdomen) were not. In fact, the counts from the inter-sampling sites did not differ significantly. Therefore, we used both the left and right sides of the abdomen as test sites. To reduce the variation in baseline bacterial counts and to ensure the baseline bacterial counts were sufficient to evaluate the bactericidal effects of antiseptics, only animals having bacterial counts of 4.0 to 5.5 log_10_ (c.f.u. cm^−2^) in the groin were used.

The bacteria isolated from the plates used to determine the baseline viable bacterial counts in experiment 1 were identified. All 144 colonies were Gram-positive (cocci, 97.2 %, 140 colonies; bacilli, 2.8 %, 4 colonies) and catalase-positive (Table 1). The bacteria reflected the common bacterial flora on the skin of animals [18–21].

**Table 1.** Skin flora on the abdomens of cynomolgus monkeys Results of analysing 144 colonies.

Gram strain	(%)		Morphotype	(%)		Genus	(%)		Species	(%)
Positive	100.0		Coccus	97.2		*Staphylococcus*	73.6		*Staphylococcus intermedius*	31.3
									*Staphylococcus sciuri*	12.5
									*Staphylococcus cohnii* ssp. *cohnii*	6.3
									*Staphylococcus capitis*	4.2
									*Staphylococcus hominis*	4.2
									*Staphylococcus cohnii* ssp*. urealyticus*	3.5
									*Staphylococcus haemolyticus*	2.8
									*Staphylococcus carnosus*	1.4
									*Staphylococcus lentus*	1.4
									*Staphylococcus saprophyticus*	1.4
									*Staphylococcus xylosus*	1.4
									*Staphylococcus auricularis*	0.7
									*Staphylococcus caprae*	0.7
									*Staphylococcus hyicus*	0.7
									*Staphylococcus lugdunensis*	0.7
									*Staphylococcus warneri*	0.7
						*Kocuria*	14.6		*Kocuria varians/rosea*	13.9
									*Kocuria kristinae*	0.7
						*Micrococcus*	9.0		*Micrococcus* spp.	9.0
			Bacillus	2.8		*Corynebacterium*	2.1		*Corynebacterium auris/Turicella otitidis*	0.7
									*Corynebacterium propinquum*	0.7
									*Corynebacterium renale* group	0.7
						*Rothia*	0.7		*Rothia dentocariosa*	0.7

### Bactericidal effects in clean condition

**Fig. 2.**
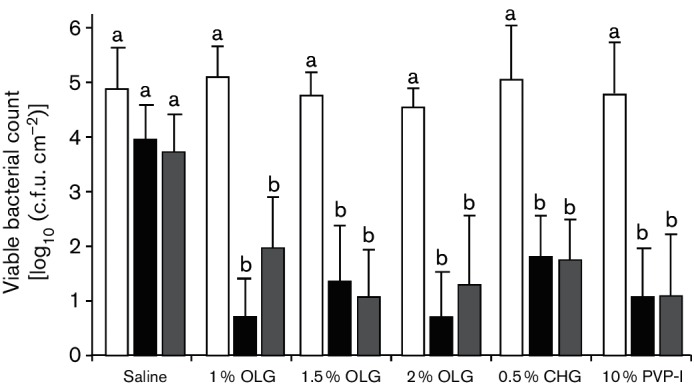
Changes in viable bacterial counts from baseline (white columns) to 10 min (black columns) and to 6 h (grey column) after antiseptic application against skin flora of cynomolgus monkeys in clean condition. Each column and vertical bar represents the mean and sd for eight test sites. Values not sharing a common letter at same time point differ significantly at *P*<0.05 by Tukey’s multiple comparisons test.

The bactericidal effects of 1.0, 1.5 and 2 % OLG applied to the normal skin without extra treatments were compared to those of 0.5 % CHG, 10 % PVP-I, and saline (as a negative control). Baseline bacterial counts did not differ significantly between groups (Fig. 2). After 10 min (the fast-acting effect), the bacterial counts of all antiseptic-groups were significantly lower than that of saline but did not differ significantly among themselves (Fig. 2). The log_10_ reductions of all antiseptic-groups were also significantly higher than those of saline, but again did not differ significantly among themselves (Table 2). A clear dose-dependency of OLG was also not noted. In three test sites in the 1 % OLG group, two in the 2 % OLG group, and three in the PVP-I group of eight test sites, the bacterial counts decreased to zero, suggesting that the log_10_ reductions may have been underestimated.

**Table 2.** Log_10_ reductions of skin flora in cynomolgus monkeys after application of antiseptics

Test antiseptic	Log_10_ reduction in bacterial count (mean±sd, *n*=8)	
	10 min after application	6 h after application
Saline	0.92±0.30^a^	1.15±0.27^a^
1 % Olanexidine gluconate	4.38±0.87^b^	3.13±0.86^b^
1.5 % Olanexidine gluconate	3.41±1.23^b^	3.70±0.77^b^
2 % Olanexidine gluconate	3.84±0.56^b^	3.23±0.98^b^
0.5 % Chlorhexidine gluconate	3.24±0.93^b^	3.31±0.43^b^
10 % Povidone–iodine	3.71±0.86^b^	3.69±1.11^b^

Values not sharing a common letter at same time point differ significantly at *P*<0.05 by Tukey’s multiple comparisons test.

The pattern of bacterial counts at 6 h after application (long-lasting effect) was similar to that after 10 min (Fig. 2). The log_10_ reductions were significantly higher than that in saline, but did not differ significantly among antiseptics (Table 2). At this time point, a clear dose-dependency of OLG was also not noted. In one test site each of the 1.5 and 2 % OLG groups and three in the PVP-I group of eight test sites, the viable bacterial counts decreased to zero, suggesting that the log reductions may have been underestimated.

### Bactericidal effects in dirty conditions

To evaluate the bactericidal effects of the test antiseptics when the antiseptics were applied to the skin in dirty conditions, blood was used as a contaminant in two experiments. In the first experiment, the bactericidal effects of the test antiseptics were examined on the skin contaminated with blood, which is considered to be a model of emergency patients with traumatic injury (experiment 2). In the second experiment, the bactericidal effects were examined on the skin contaminated with blood after application of antiseptic, which is considered to be a model of patients bleeding after skin preparation (experiment 3). In experiment 1, no clear dose-dependency of OLG was observed; therefore, the next two experiments (experiments 2 and 3) were conducted using the middle dose, 1.5 %.

In these experiments, application materials (equipment) for OLG application and CHG or PVP-Iapplication were sponge and cotton ball, respectively. OLG was applied with sponges because OLG was planned to be marketed as an antiseptic applicator product which consists of the drug solution, sponge foam and handle. To determine whether the difference of application materials affected bactericidal activity, we tested PVP-I in the clean condition. When PVP-I was applied to eight sites by cotton balls, the log_10_ reductions (mean±sd) were 3.20±1.10 at 10 min and 3.43±0.65 at 6 h. When PVP-I was applied with sponges, the log_10_ reductions were 3.71±0.86 at 10 min and 3.69±1.11 at 6 h (Table 2). At neither time point did these bactericidal effects differ significantly by method of application based on an analysis of covariance. In addition, the 95 % CIs of the difference between the sponge application and cotton ball application were −0.58 to 1.52 and −0.78 to 1.16 at 10 min and 6 h after the application, respectively, which include zero. Therefore, it was considered that the difference of the application materials did not affect the log_10_ reduction.

**Fig. 3.**
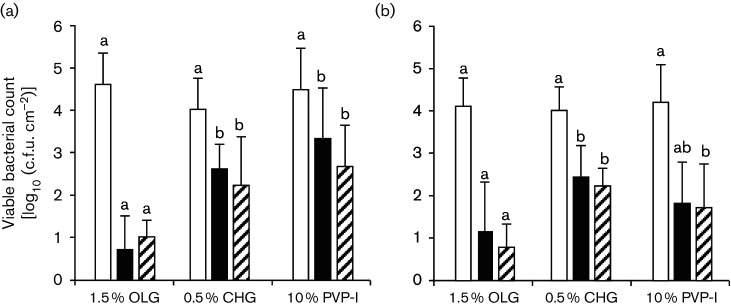
Changes in viable bacterial counts from baseline (white column) to 10 min (black column) to 6 h (hatched column) after antiseptic application on the skin flora of cynomolgus monkeys in the contaminated condition; blood contamination before antiseptic applied (a) and blood contamination after antiseptic applied (b). Each column and vertical bar represents the mean and sd for eight test sites. Values not sharing a common letter at same time point differ significantly at *P*<0.05 by Tukey’s multiple comparisons test.

In the condition of blood contamination before antisepsis (experiment 2), baseline viable bacterial counts did not differ significantly among groups (Fig. 3a). The log_10_ reductions of 1.5 % OLG at both time points were significantly higher than those of 0.5 % CHG and 10 % PVP-I (Table 3). In this condition, both the fast-acting and long-lasting bactericidal effects of 1.5 % OLG were significantly stronger than those of 0.5 % CHG and 10 % PVP-I.

**Table 3.** Log_10_ reductions in skin flora of cynomolgus monkeys after application of antiseptics before and after blood contamination

Test antiseptic	Log_10_ reduction in bacterial count (mean±sd, *n*=8)				
	Blood contamination before antiseptic applied			Blood contamination after antiseptic applied	
	10 min after application	6 h after application		10 min after application	6 h after application
1.5 % Olanexidine gluconate	3.90±0.41^a^	3.58±0.77^a^		2.96±0.90^a^	3.33±0.57^a^
0.5 % Chlorhexidine gluconate	1.38±0.70^b^	1.80±0.95^b^		1.56±0.54^b^	1.79±0.57^b^
10 % Povidone–iodine	1.15±0.55^b^	1.83±0.71^b^		2.40±0.72^ab^	2.48±0.67^b^

Values not sharing a common letter at same time point differ significantly at *P*<0.05 by Tukey’s multiple comparisons test.

In the condition of blood contamination after antisepsis (experiment 3), baseline viable bacterial counts did not differ significantly among groups (Fig. 3b). The log_10_ reductions of 1.5 % OLG were again significantly higher than those of CHG at both time points and higher than those of PVP-I at 6 h after application (Table 3). In this condition, the fast-acting and long-lasting bactericidal effects of 1.5 % OLG were significantly stronger than those of 0.5 % CHG and equal to or stronger than those of 10 % PVP-I.

## Discussion

The efficacy of antiseptics has been determined exclusively by *in vitro* tests. For the evaluation of the new antiseptic which cannot be tested in humans and the clinical studies considering clinical use, *in vivo* model using cynomolgus monkeys whose body size is easy to handle is considered to be useful.

Animal skin flora mainly consists of micrococci, coagulase-negative staphylococci (CNS) and corynebacteria [22]. For example, *Micrococcus* spp., CNS, *Clostridium* spp., *Propionibacterium acnes*, *Acinetobacter* spp. and various Gram-negative aerobes are considered normal residents of the surface of dog skin [19, 23]. The resident flora of cats includes *Micrococcus* spp., CNS and *Acinetobacter* spp. [23, 24]. *Bacillus* spp., *Staphylococcus* spp., *Micrococcus* spp. and *Corynebacterium* spp. are found on the skin of farm animals, such as horses, sheep and cattle [25–28]. In our study, cynomolgus monkeys had bacterial skin flora similar to that of these other animals. Gram-positive bacterial species were predominantly detected under the aerobic culture conditions (Table 1). Also, the bacterial skin flora of cynomolgus monkeys were not considered to be markedly different from those on human skin. Under normal breeding conditions, cynomolgus monkeys also have sufficient numbers of bacteria on the skin to evaluate the efficacy of an antiseptic. In humans, the abdomen and groin are recognized as representatives of a dry site and a moist site, respectively, and the bacterial counts at the groin are correspondingly higher than those in the abdomen. In cynomolgus monkeys, bacteria were uniformly distributed on the skin, and both abdomen and groin are considered to be dry sites.

In this test system, we determined the bactericidal effect of a new antiseptic, OLG. We considered the statistical power of the primate model as follows. From the results of antiseptic application in 56 test sites, including preliminary experiments and the experiments described in this paper, the common standard deviation of log_10_ reduction was 0.94. For Student’s *t*-test under the conditions of α=0.05, β=0.2 and sample size=8, we had an 80 % chance of detecting a difference in log_10_ reduction of 1.5.

In clean conditions (experiment 1), the log_10_ reduction of physiological saline, which has no bactericidal effect, was approximately 1 log_10_ (Table 2). This result is thought to be caused by a washout effect. Obviously, the log_10_ reduction of OLG, CHG and PVP-I was significantly higher than that of physiological saline (Table 2). In this condition which mimics presurgical antisepsis, the fast-acting and long-lasting bactericidal effects of OLG with a concentration of 1 % or higher were equivalent to those of 0.5 % CHG and 10 % PVP-I. In the blood-contaminated experiments, the bactericidal effects of OLG with a middle dose (1.5 %) were compared with those of CHG and PVP-I.

In both the dirty conditions, one of which was blood contamination prior to antisepsis and the other was blood contamination after antisepsis, the bactericidal effect of 1.5 % OLG was significantly stronger than that of 0.5 % CHG and equivalent to or stronger than that of 10 % PVP-I. The bactericidal effect of OLG was equivalent in clean and dirty conditions. Thus, its bactericidal effect is relatively unaffected by organic substances. This may be related the affinity of OLG to bacterial membrane being stronger than that of CHG [7].

In conclusion, the primate model is considered to be an effective model which can be used to test the efficiency of antiseptics. The advantages of this model are as follows: (i) suitable size to handle; (ii) appropriate for a pilot study of the new antiseptic which cannot be tested on humans; (iii) appropriate for the evaluation of the effect against the resident skin flora; and (iv) enables animal study considering the clinical application method, specific clinical conditions such as skin contaminated with blood, etc. Also, a new antiseptic, OLG, has bactericidal effects in this model. The efficacy of OLG was comparable to that of commercial antiseptic formulations such as CHG and PVP-I in clean conditions. But in the blood-contaminated conditions, OLG is more effective than the commercial formulations and its bactericidal effect is hardly affected by organic substances. Therefore, it is considered that OLG has a favourable property for skin preparation in various types of surgical treatments.
